# Perovskite LaNiO_3_/Ag_3_PO_4_ heterojunction photocatalyst for the degradation of dyes

**DOI:** 10.3389/fchem.2022.969698

**Published:** 2022-12-08

**Authors:** Shahzad Ameen, Maida Murtaza, Muhammad Arshad, Aiyeshah Alhodaib, Amir Waseem

**Affiliations:** ^1^ Department of Chemistry, Quaid-i-Azam University, Islamabad, Pakistan; ^2^ Nanosciences and Technology Department, National Centre for Physics (NCP), Quaid-i-Azam University (QAU) Campus, Islamabad, Pakistan; ^3^ Department of Physics, College of Science, Qassim University, Buraydah, Saudi Arabia

**Keywords:** water pollution, photocatalysis, silver phosphate, lanthanum nickelate, dyes

## Abstract

Pristine lanthanum nickelate (LaNiO_3_), silver phosphate (Ag_3_PO_4_) and perovskite lanthanum nickelate silver phosphate composites (LaNiO_3_/Ag_3_PO_4_) were prepared using the facile hydrothermal method. Three composites were synthesized by varying the percentage of LaNiO_3_ in Ag_3_PO_4_. The physical properties of as-prepared samples were studied by powder X-ray diffraction (pXRD), Fourier-transform infrared (FT-IR), Scanning electron microscopy (SEM) and Energy-dispersive X-ray (EDX). Among all synthesized photocatalysts, 5%LaNiO_3_/Ag_3_PO_4_ composite has been proved to be an excellent visible light photocatalyst for the degradation of dyes i.e., rhodamine B (RhB) and methyl orange (MO). The photocatalytic activity and stability of Ag_3_PO_4_ were also enhanced by introducing LaNiO_3_ in Ag_3_PO_4_ heterojunction formation. Complete photodegradation of 50 mg/L of RhB and MO solutions using 25 mg of 5%LaNiO_3_/Ag_3_PO_4_ photocatalyst was observed in just 20 min. Photodegradation of RhB and MO using 5%LaNiO_3_/Ag_3_PO_4_ catalyst follows first-order kinetics with rate constants of 0.213 and 0.1804 min^−1^, respectively. Perovskite LaNiO_3_/Ag_3_PO_4_ photocatalyst showed the highest stability up to five cycles. The photodegradation mechanism suggests that the holes (***h*** ^+^) and superoxide anion radicals $\left.{\left(O\right.}_{2}^{\;•-}\right)$ plays a main role in the dye degradation of RhB and MO.

## Introduction

The increase in the usage of inorganic and organic substances and the surge in industrialization and overpopulation have adverse effects on the soil and the quality of freshwater resources in recent years. In our daily lives, dyes are frequently used, as well as their contact with the environment is detrimental because of their risk inherent to plants, humans and wildlife (Chowdhary et al., 2020).

Water is a vital resource for human growth and life on Earth. Textile manufacturing is one of the anthropogenic processes that pollute water sources. Synthetic dyes are utilized in considerable amounts in various industries. Pharmaceutical, paint manufacturing, cosmetics, leather and clothing industries discharge tons of various kinds of dyes into the environment (Waseem et al., 2014). During the coloring of fabric, roughly 10–15% of azo dyes do not bind and are discharged into water bodies. That’s why azo dyes show more health concerns than non-azo dyes. Azo dyes are toxic for microbial communities as well as plant growth and germination. Most of these dyes are potentially non-biodegradable and extremely stable, resulting in serious environmental impacts on marine life and humans (Markandeya et al., 2017; Lellis et al., 2019).

Non-biodegradable MO is one of the common azo dyes is carcinogenic and harmful compound, if ingested, it might cause irritation of the respiratory tract, eyes and skin (Aziztyana et al., 2019; Lellis et al., 2019). In an alkaline medium, MO appears yellow, but in an acidic one, it appears red. Rhodamine B dye is commonly used in industries and is a non-biodegradable dye, causes numerous environmental contamination issues by releasing carcinogenic and harmful compounds into the water. If RhB is ingested, it might cause irritation of the abdominal disorders, blindness, respiratory distress and skin sensitization. (Markandeya et al., 2017; Areeb et al., 2021). In an alkaline medium, RhB appears colorless, but in an acidic one, it appears pink. Methyl orange is a type of azo dye that is commonly used in textiles.

Due to their high-water solubility, dyes and organic chemicals such as dyes are difficult to remove using traditional methods. To decompose dyes and other organic substances in water, different treatment techniques have been used. Toxins are extracted from wastewater in different ways by each approach. Major methods include 1) Chemical methods such as chlorination and ozonation, 2) Physical methods such as ultra-filtration, adsorption, reverse osmosis and ion exchange, 3) Biological methods such as aerobic as well as anaerobic treatments (Maheshwari et al., 2021). The majority of the traditional approaches just change the phase of chemicals without transforming them into eco-friendly products. As a result, their application is not compatible since they produce harmful side products that require additional treatment, bringing excessive expense to the degradation process (Katheresan et al., 2018).

Photoreactions of semiconductors due to their greater potential to mineralize organic pollutants in the environment have become highly significant among the various Advanced Oxidation Processes (AOPs) in recent years (Szczepanik, 2017). Among the numerous novel technologies accessible today, the use of materials for heterogeneous photocatalysis/electrocatalysis has been regarded as one of the most efficient methods for tackling both energy as well as environmental challenges and has thus gained a lot of attention in recent years (Gao et al., 2021; Wu et al., 2021). Due to their good magnetic, physical and optical properties, a number of nanostructured semiconductors such as ZnS, ZnO, CdS, TiO_2_ and WO_3_ are used as photocatalysts (Sudha and Sivakumar, 2015; Luo et al., 2019; Saravanan et al., 2020). Under visible light, silver orthophosphate (Ag_3_PO_4_) has a very strong photocatalytic activity. According to charge neutrality analysis, Ag_3_PO_4_ may act as a weakly p-type semiconductor for O-rich conditions and as a weakly n-type semiconductor for O-poor conditions (Reunchan and Umezawa, 2013), and has a band gap of 2.36 eV semiconductor with good visible light degradation of organic pollutants and induced oxygen evolution (Li et al., 2019). However, due few disadvantages of silver phosphate such as: photogenerated electrons can easily reduce Ag^+^ in Ag_3_PO_4_ to elemental silver (Ag^0^), and electron-hole pair recombination. (Zhang et al., 2014; Chen et al., 2022). The composite formation is an effective method for expanding absorption from ultraviolet to visible region that either adds a new band into the initial band or alters the valence band (VB) or conduction band (CB), improving photocatalytic activity (Zhang et al., 2017; Lv et al., 2019). Composite formation of Ag_3_PO_4_ with other materials such as carbon nanotubes (Xu et al., 2014), graphene oxide (Chen G. et al., 2013) have also been reported in recent years. Similarly, combining other semiconductors with Ag_3_PO_4_ to form a heterojunction is also a promising strategy. For example, Bi_4_Ti_3_O_12_/Ag_3_PO_4_ (Zheng et al., 2017), TiO_2_/Ag_3_PO_4_ (Liu et al., 2019), ZnWO_4_/Ag_3_PO_4_ (Zhang et al., 2017), BiVO_4_/Ag_3_PO_4_ (Qi et al., 2016) *etc.* The advantages of coupled semiconductors include extending the light responsive range and boosting the transferring abilities of the photo-generated charge carrier.

In this study, the stability and photocatalytic activity of Ag_3_PO_4_ is enhanced by composite formation with LaNiO_3,_ ABO_3_ type perovskite. Perovskites are another appealing semiconductor photocatalyst with changeable components, simple structure, and good stability (Grabowska, 2016; Zhang et al., 2016). The theoretical bandgap energy (2.40 eV) of LaNiO_3_ allows it to absorb visible light energy, but in actual practice, the photocatalytic activity of pure LaNiO_3_ is not as good as expected due to poor response to visible light and electron and hole pair recombination (Gao et al., 2019). Combining LaNiO_3_ with Ag_3_PO_4_ shows a facile way to improve photocatalytic activity and stability for water treatment.

## Experimental

### Materials

Sodium hydroxide (NaOH), Lanthanum nitrate hexahydrate (La(NO_3_)_3_ ·6H_2_O) and Nickel nitrate hexahydrate (Ni(NO_3_)_2_ ·6H_2_O) were purchased from Sigma Aldrich. ethanol (CH_3_CH_2_OH) was obtained from Merk (Germany). Silver nitrate (AgNO_3_) and Sodium phosphate (Na_3_PO_4_) were provided by Sigma-Aldrich. All the chemicals were of analytical grade, and all the solutions were prepared in distilled water.

### Synthesis of perovskite LaNiO_3_

Perovskite LaNiO_3_ was prepared by the facile hydrothermal method. In a typical synthesis, stoichiometry amounts of lanthanum nitrate hexahydrate and nickel nitrate hexahydrate were mixed in distilled water under stirring conditions. Sodium hydroxide was added to maintain the pH 11. The resulting mixture was transferred into an autoclave for hydrothermal treatment at 180°C for 12 h. The product was washed with distilled water and ethanol and dried overnight. Then the product was calcined at 650°C for 4 hours in order to obtain the lanthanum nickelate powder.

### Synthesis of perovskite LaNiO_3_/Ag_3_PO_4_ composites

Composites of lanthanum nickelate and silver phosphate were prepared by the hydrothermal method (Guan and Guo, 2014). In a typical procedure, an appropriate amount of lanthanum nickelate was dispersed in distilled water by sonication, stoichiometry amounts of silver nitrate and sodium phosphate were added in the mixture. The yellow precipitates of silver phosphate were observed. Immediately the mixture was transferred into Teflon lined autoclave for 24 h at 100°C. The obtained composite was washed with water and ethanol. Three composites were obtained by varying the amounts of lanthanum nickelate and labeled as 2% LaNiO_3_/Ag_3_PO_4_, 5% LaNiO_3_/Ag_3_PO_4_ and 10% LaNiO_3_/Ag_3_PO_4_. Pure silver phosphate was prepared by same procedure without adding lanthanum nickelate.

### Procedure for the measurement of catalytic performance

The efficiency of as prepared photocatalysts was examined by the photodegradation of RhB and MO under visible light (using 100 watts LED light with an output of 40 k Lux, measured with Extech LT300 light meter). In a typical procedure, 0.025 g of LaNiO_3_/Ag_3_PO_4_ photocatalyst is added to the solution of RhB and MO (25 ppm) under stirring conditions using a magnetic stirrer at room temperature. Suspensions were collected at given time intervals, and the photocatalyst was removed with the help of a centrifuge (4,000 rpm, 5 min). UV–Vis spectrophotometer (Model UV-1700 SHIMADZU) was used to determine the concentration of residual RhB and MO in supernatant solution depending on the highest absorption of RhB and MO at wavelengths of 554 and 464 nm, respectively. Degradation efficiency was calculated using the following relationship.

$$DegradationEfficiency\left(\%\right)=\frac{\left(C₀-Ct\right)}{C₀}\times 100,$$ (1)

Where C_t_ is concentration after time t and C_o_ is the initial concentration.

## Results and discussion

### Powder X-ray diffraction analysis

**FIGURE 1:**
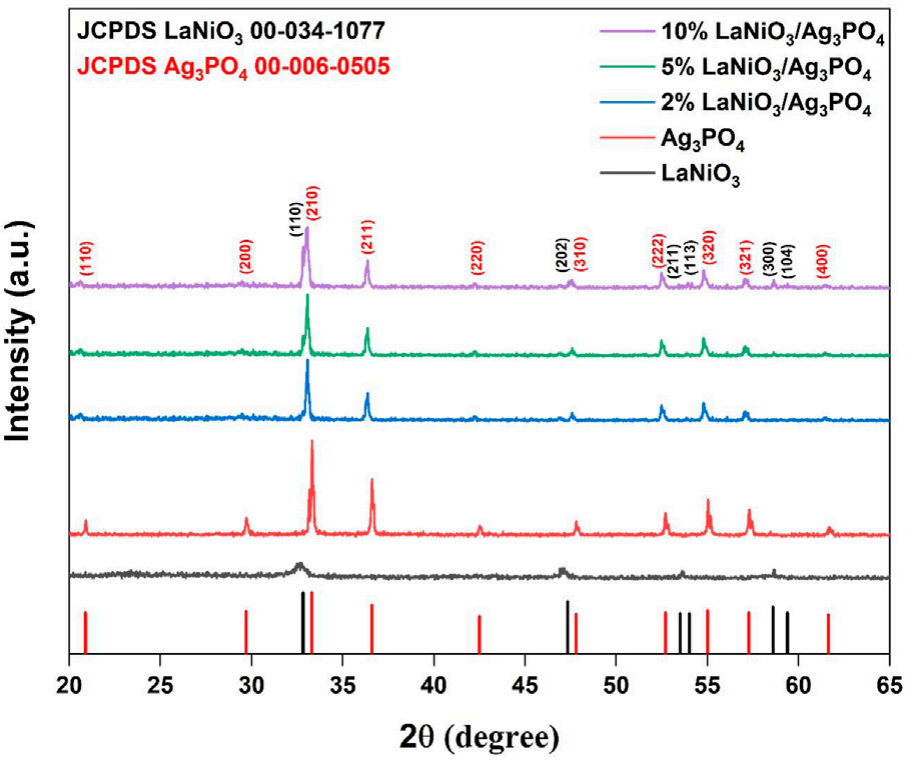
Powder XRD pattern of LaNiO_3_, Ag_3_PO_4_, 2% LaNiO_3_/Ag_3_PO_4_, 5% LaNiO_3_/Ag_3_PO_4_ and 10% LaNiO_3_/Ag_3_PO_4_ composite.

The diffraction pattern of LaNiO_3_ shows 2theta values of 32.8°, 47.3°, 53.5°, 54.1°, and are well matched with the rhombohedral phase of LaNiO_3_ (JCPDS No.00-034-1077; space group, R), while those of Ag_3_PO_4_ corresponded to 2theta values of 20.9°, 29.7°, 33.2°, 36.5°, 47.7°, 52.7°, 57.1° shows the cubic structure of Ag_3_PO_4_ (JCPDS No.00-006-0505; space group, P4̅ 3n). The XRD pattern of LaNiO_3_/Ag_3_PO_4_ composites showed a combination of LaNiO_3_ and Ag_3_PO_4_ and ruled out the possibility of other impurity phases, indicating successful synthesis of composite. Furthermore, it is observed that the diffraction peaks of LaNiO_3_ gradually strengthened with increasing the LaNiO_3_ content, while the peak intensities of Ag_3_PO_4_ weakened. The hydrothermal treatment gave well-crystallized Ag_3_PO_4_ particles with sharp diffraction peaks. Compared to Ag_3_PO_4_, peaks of LaNiO_3_ were broader and less sharp, which may be resulting from smaller crystallite size (Figure 1). The crystallite size of 14.7 nm, 86.5 nm, 58.1 nm were calculated for LaNiO_3_, Ag_3_PO_4_ and 5% LaNiO_3_/Ag_3_PO_4_ composite respectively using Scherrer equation.

### FT-IR spectroscopy

**FIGURE 2:**
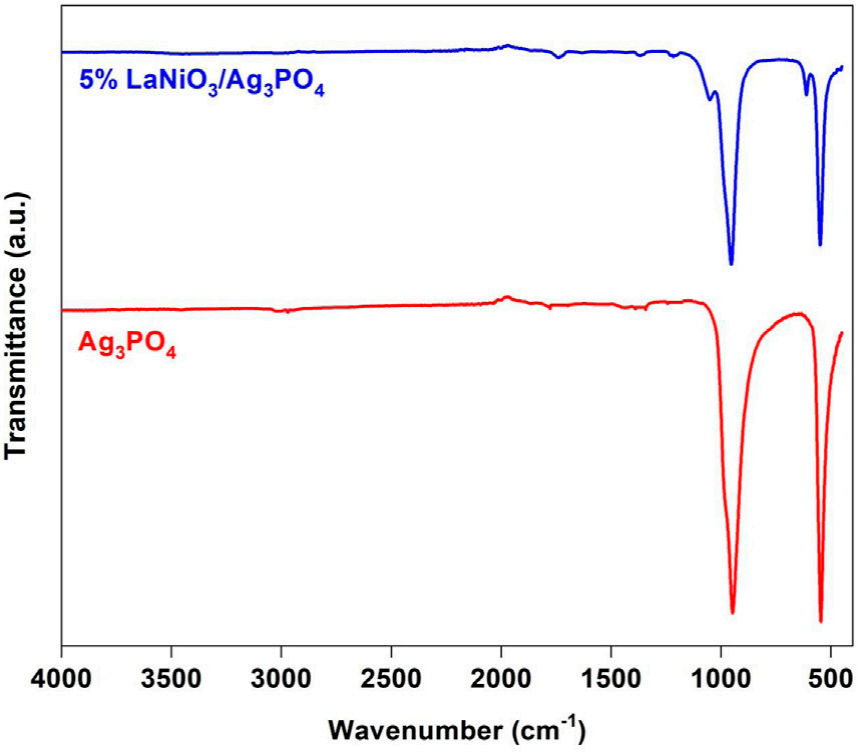
FT-IR spectra of Ag_3_PO_4_ and 5% LaNiO_3_/Ag_3_PO_4_.

Figure 2 shows the FT-IR spectra of Ag_3_PO_4_ and 5% LaNiO_3_/Ag_3_PO_4_. In the spectrum of silver phosphate, two strong peaks were observed at 550 cm^−1^ and 946 cm^−1^ and were assignable to bending and stretching vibrations of the P-O bond, respectively. Two small peaks at 565 cm^−1^ and 965 cm^−1^ is observed for bending vibrations of Ni-O bond.

### Scanning electron microscopy

**FIGURE 3:**
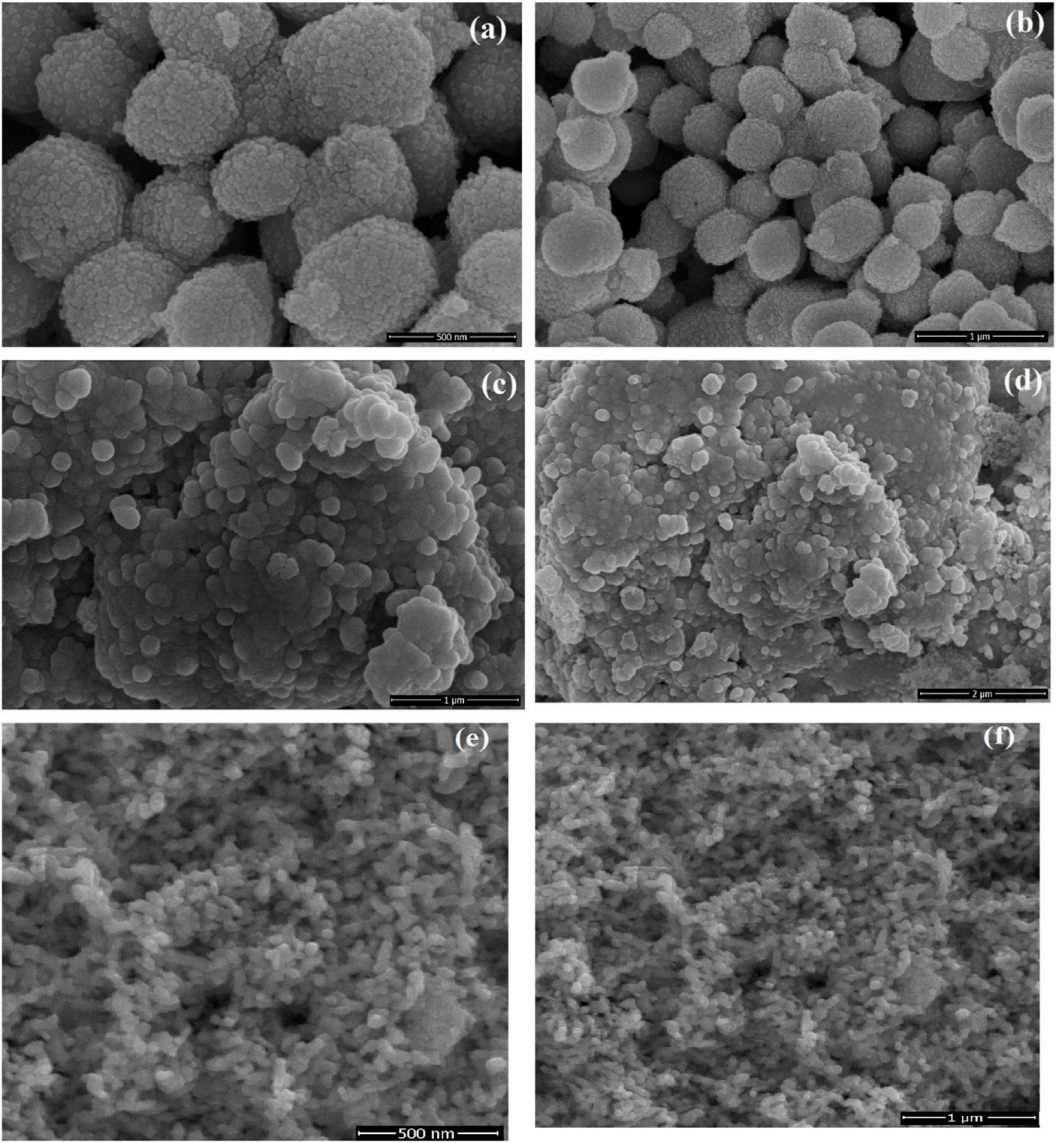
SEM images of Ag_3_PO_4_ **(A,B)**, and LaNiO_3_/Ag_3_PO_4_ composite **(C,D)**, LaNiO_3_ **(E,F)** at different resolutions.

SEM was used to investigate the morphology and particle size of as-prepared samples. SEM images of 5% LaNiO_3_/Ag_3_PO_4_ showed that particles of LaNiO_3_ were uniformly and tightly attached on the surface of Ag_3_PO_4_, which indicated an intimate contact between LaNiO_3_ and Ag_3_PO_4_. Pure Ag_3_PO_4_ possessed a polyhedral morphology and has interstitial spaces, as shown in Figures 3A,B, whereas, these spaces were filled with LaNiO_3_ accumulation in 5% LaNiO_3_/Ag_3_PO_4_ composite. Figures 3E,F shows the very tiny particles of LaNiO_3_. Figures 3C,D showed that LaNiO_3_ particles are uniformly distributed on the surface of Ag_3_PO_4_.

### Energy-dispersive X-ray spectroscopy

**FIGURE 4:**
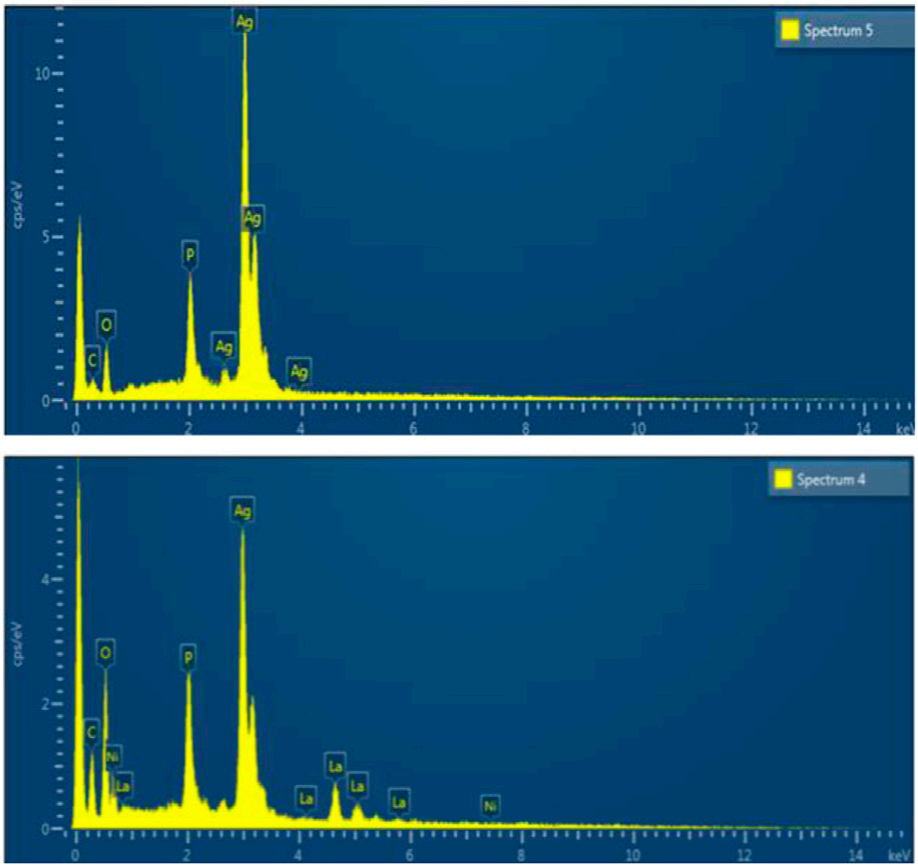
EDX spectrum of (upper) Ag_3_PO_4_ (lower) 5%LaNiO_3_.

EDX was used to find the elemental composition of Ag_3_PO_4_ and LaNiO_3_/Ag_3_PO_4_ composite. Figure 4 depicted the elemental composition of Ag_3_PO_4_ and 5%LaNiO_3_/Ag_3_PO_4_ composite. The EDX Ag_3_PO_4_ shows (wt%) Ag = 77, *p* = 7.3, O = 15.7, whereas 5%LaNiO_3_/Ag_3_PO_4_ shows (wt%) Ag = 73.1, *p* = 6.9, La = 2.9, Ni = 1.2, O = 15.8. The energy-dispersive X-ray spectrum of composite shows that La, Ni, Ag, P, and O are present in the composite and confirmed the successful synthesis of the composite.

### Band gap estimation and optical study

**FIGURE 5:**
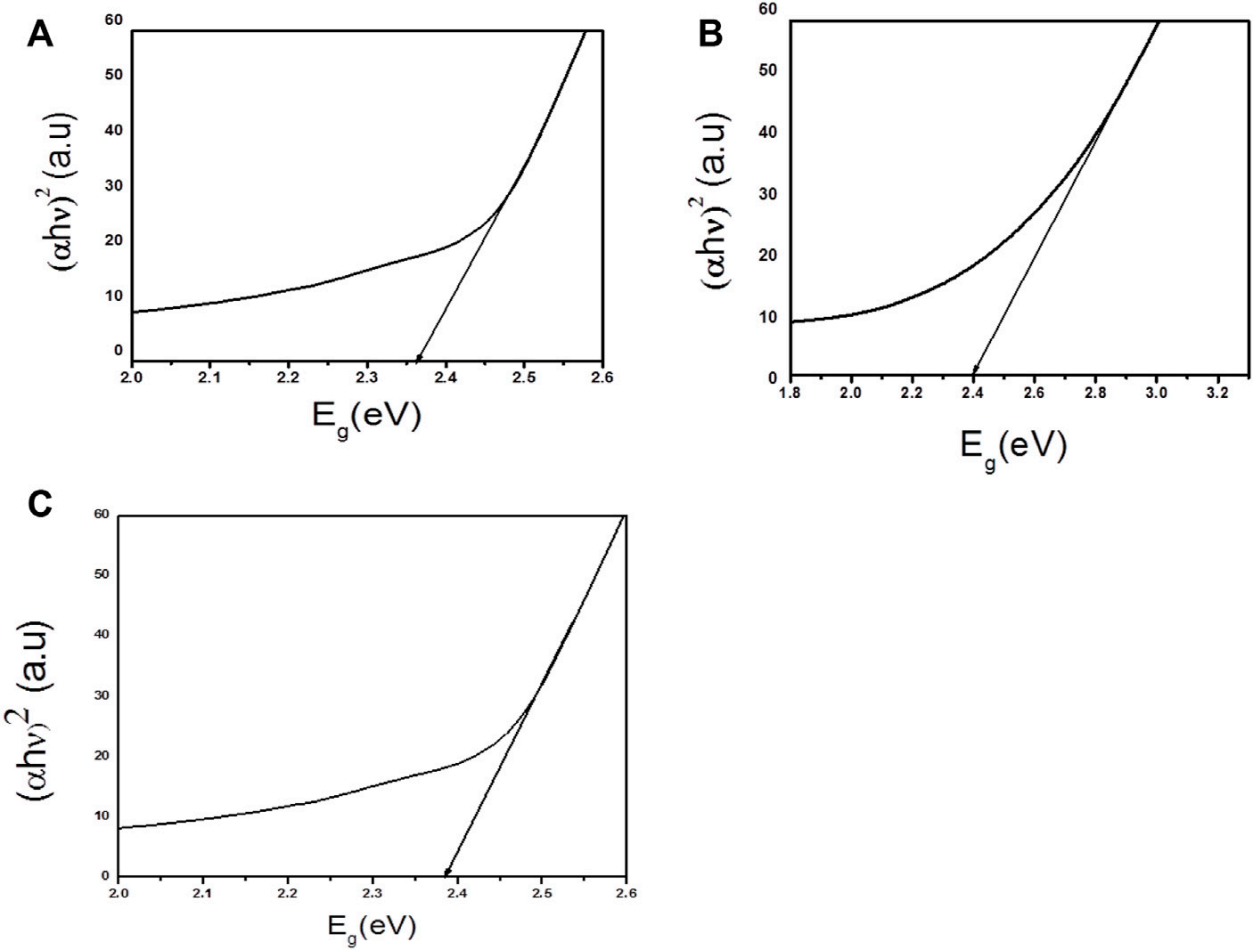
Tauc plots of **(A)** Ag_3_PO_4_ **(B)** LaNiO_3_ **(C)** 5% LaNiO_3_/Ag_3_PO_4_.

When light falls on the surface of semiconductor photocatalyst electrons move from VB to CB by absorbing energy. The relationship between the band gap energy and absorption spectra can be represented by Tauc equation which is written as:

$$\left(\alpha h\upsilon \right)=k{\left(h\upsilon -E_{g}\right)}^{n}$$

where ***hυ*** is the energy of photon, n is a type of transition, and n is either 1/2 for a direct transition or 2 for an indirect transition, k is the band tailing parameter, that does not dependent on energy, and E_g_ is the energy of band gap (Masnadi-Shirazi et al., 2014). The Beer-Lambert law can be used to calculate coefficient of absorption (α). Band gap value (E_g_) between the wavelength ranges from 200 to 800 nm can be quantified by extrapolating linear region of plot energy (hυ) vs (αhυ)^2^. Different parameters such as doping, grain size, annealing treatment and type of transition (direct or indirect), create differences in Eg values (Hameeda et al., 2021). The band gap energies of Ag_3_PO_4_ and LaNiO_3_ were found to be 2.36 and 2.40 eV respectively, which are similar to previous findings (Li et al., 2019; Chen et al., 2022). These photocatalysts have comparable band gap energies. The band gap energy of 5% LaNiO_3_/Ag_3_PO_4_ composite is 2.38 eV. Tauc plots of Ag_3_PO_4_, LaNiO_3_ and 5% LaNiO_3_/Ag_3_PO_4_ are depicted in the Figures 5A–C respectively.

### Optimization studies for rhodamine B photodegradation

The purpose of this work is to investigate new avenues in the study of photodegradation efficiency of highly effective and freshly synthesized photocatalysts for rhodamine B (RhB) degradation using various parameters such as pH, catalyst dose, time and concentration of dye. The effect of dye adsorption in dark was also studied and it was observed that no significant adsorption was observed in the absence of light.

**FIGURE 6:**
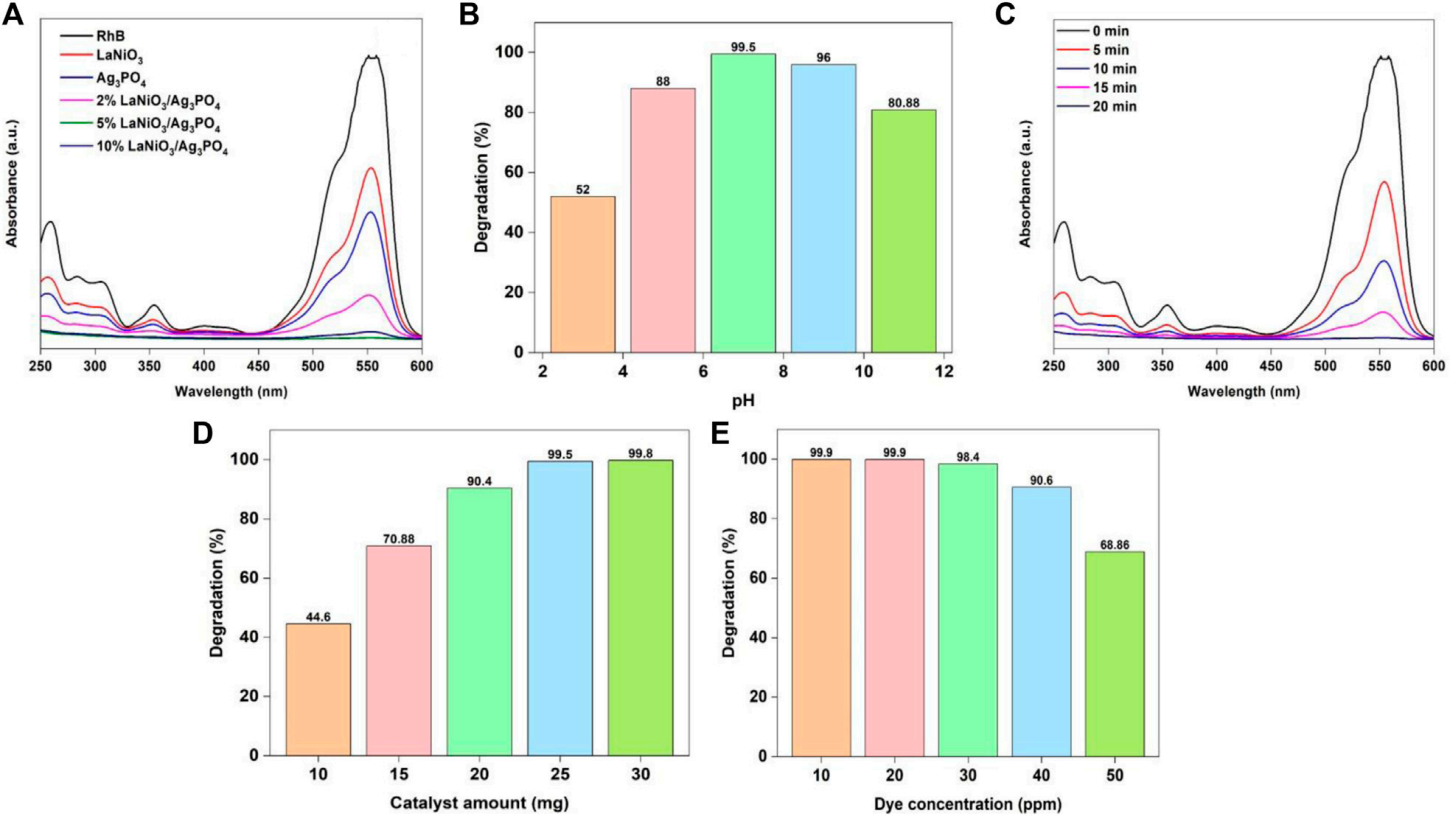
Change of RhB degradation efficiency of photocatalysts with **(A)** type of catalyst, **(B)** pH **(C)** time, **(D)** catalyst amount **(E)** dye concentration. Reaction conditions: Light Source: 100 watts LED, Intensity: 40 k Lux, temperature: 25°C, Pressure: 1 atm.

The photocatalytic activity of as-prepared catalysts for rhodamine B degradation was investigated with UV-Visible spectrophotometry, by taking 25 mg of the each photocatalyst and 30 ml of RhB solution (25 mg/L). The resultant solutions were exposed to light lasting 20 min while being continuously magnetically stirred. The most appropriate catalyst was selected based on their performance for dye degradation. The absorption spectrum of RhB from photocatalytic studies was observed between the wavelength ranges from 200 to 600 nm. After a specified period of time, the strength of the RhB distinctive peak began to decrease (Figure 6A). The optimum catalyst was selected depending on the efficiency of dye degradation. The photocatalytic activity increases by increasing the content of LaNiO_3_ in the LaNiO_3_/Ag_3_PO_4_ composite up to 5% but it decreases by further increasing the LaNiO_3_ content due to the possible formation of agglomeration. The results demonstrate that the 2%LaNiO_3_/Ag_3_PO_4_ and 10%LaNiO_3_/Ag_3_PO_4_ composite showed higher activity than pure LaNiO_3_ and Ag_3_PO_4_ but lower than 5% LaNiO_3_/Ag_3_PO_4_ composite. Therefore, 5%LaNiO_3_/Ag_3_PO_4_ composite was found to be good photocatalyst for the degradation of rhodamine B providing good stability and synergy for photocatalysis.

pH of the solution is an important parameter in studying the degradation reaction. The surface charges of the photocatalyst are directly affected by pH. The RhB solution’s pH was regulated using 1 M sodium hydroxide and 1 M hydrochloric acid solution. The pH of the solution is adjusted at the commencement of the reaction. The photodegradation investigation was conducted under the conditions: (Irradiation time: 20 min, amount of catalyst: 25 mg, solution’s pH: 2–10, concentration of RhB: 25 mg/L). According to Figure 6B the degradation efficiency of RhB using 5%LaNiO_3_/Ag_3_PO_4_ photocatalyst is maximum at pH 7. The pH of solution is a major factor in photocatalytic process that occurs on the surface of a photocatalyst because it determines the surface charge properties of the photocatalyst as well as the size of aggregate particles that form. As a result, pH plays a significant influence in the reaction pathways that can lead to the degradation of dye. Photocatalytic activities are likely to emerge in the existence of catalyst through electron-hole pairs generated on the photocatalysts surface by visible light irradiation. The effect of pH on the dye degradation varies greatly depending on the photocatalyst available in the water. According to prior research, the surface of Ag_3_PO_4_ catalyst is neutral at pH 5.4. In acidic conditions, the surface of photocatalyst is positively charged, whereas in alkaline media, it is negatively charged. The highest degradation efficiency of RhB was achieved in neutral solutions (pH 7), but it was less efficient in alkaline medium. Despite being a cationic dye, rhodamine B has a low degradation in basic solutions (above pH 7), and the surface of catalyst is negatively charged at the pH above 5.4. This strange behavior can be attributed to the fact that silver ions in silver phosphate reduced to elemental silver which reduces the active site of the catalyst and degradation efficiency decreases.

Degradation of RhB with 5%LaNiO_3_/Ag_3_PO_4_ photocatalyst was examined for time optimization under visible light irradiation. Photodegradation experiment was conducted out under the conditions: (Irradiation time: 0–20 min, amount of catalyst: 25 mg, solution’s pH: 7, concentration of RhB: 25 mg/L). As seen in Figure 6C a typical absorption peak of RhB rapidly diminishes as the exposure period increases, associated with rapid change in color of dye solution. Increased exposure time to the light improves rate of electron flow from valence to the conduction band, resulting in increased degradation efficiency. After 20 min, RhB was found to have entirely degraded.

It is critical to obtain information on the optimum quantity of catalyst to be used in the reaction, not only to determine its cost, but also to ensure that it is recovered after the reaction is completed. With this in mind, the effect of catalyst quantity on RhB degradation was investigated. The photodegradation experiment was conducted out under the conditions: (Irradiation time: 20 min, amount of catalyst: 10–30 mg, solution’s pH: 7, concentration of RhB: 25 mg/L). Figure 6D depicts the influence of catalyst quantity on RhB degradation efficiency. It was found that by adding 5%LaNiO_3_/Ag_3_PO_4_ photocatalyst to reaction mixture had a significant impact on the process efficiency. The percentage of degradation rose as the catalyst dose increased, maximum at 25 mg of catalyst. The rise in the efficiency of degradation is due to the catalyst’s active sites increasing, resulting in the generation of more reactive radicals (superoxide anion and hydroxyl) that drive the degradation process.

RhB photodegradation efficiency was further studied by varying the concentrations of RhB under visible light irradiation. Photodegradation experiment was conducted out under the conditions: (Irradiation time: 20 min, amount of catalyst: 25 mg, solution’s pH: 7, concentration of RhB: 10–50 mg/L). The photodegradation reaction was shown to slow down as concentrations of RhB increased. This can happen when O_2_ adsorption is decreased due to higher dye molecules adsorption on the surface of the catalyst, resulting in a reduction of photocatalyst active sites, and thus the production of extremely oxidative ^•^O_2_^−^ is inhibited. Furthermore, high concentrations of dye cause light to self-absorb, preventing it from reaching the surface of catalyst. As a result, insufficient energy from photons reaches the catalyst’s active sites, leading to lower efficiency. In addition to that, because of the high concentration of RhB, intermediate compounds may form during the photodegradation reaction and absorb certain active radicals that may interact with the molecules of dye. Therefore, the overall degradation efficiency decreases. The degradation efficiency is still good up to 90.5% using 50 mg/L of RhB (Figure 6E).

### Optimization studies for methyl orange photodegradation

To investigate the photodegradation efficiency of highly effective and freshly synthesized photocatalysts for methyl orange (MO) degradation, various parameters such as catalyst type, pH, catalyst dose, time and dye concentration were carried out. The effect of dye adsorption in dark was also studied and it was observed that no significant adsorption was observed in the absence of light.

**FIGURE 7:**
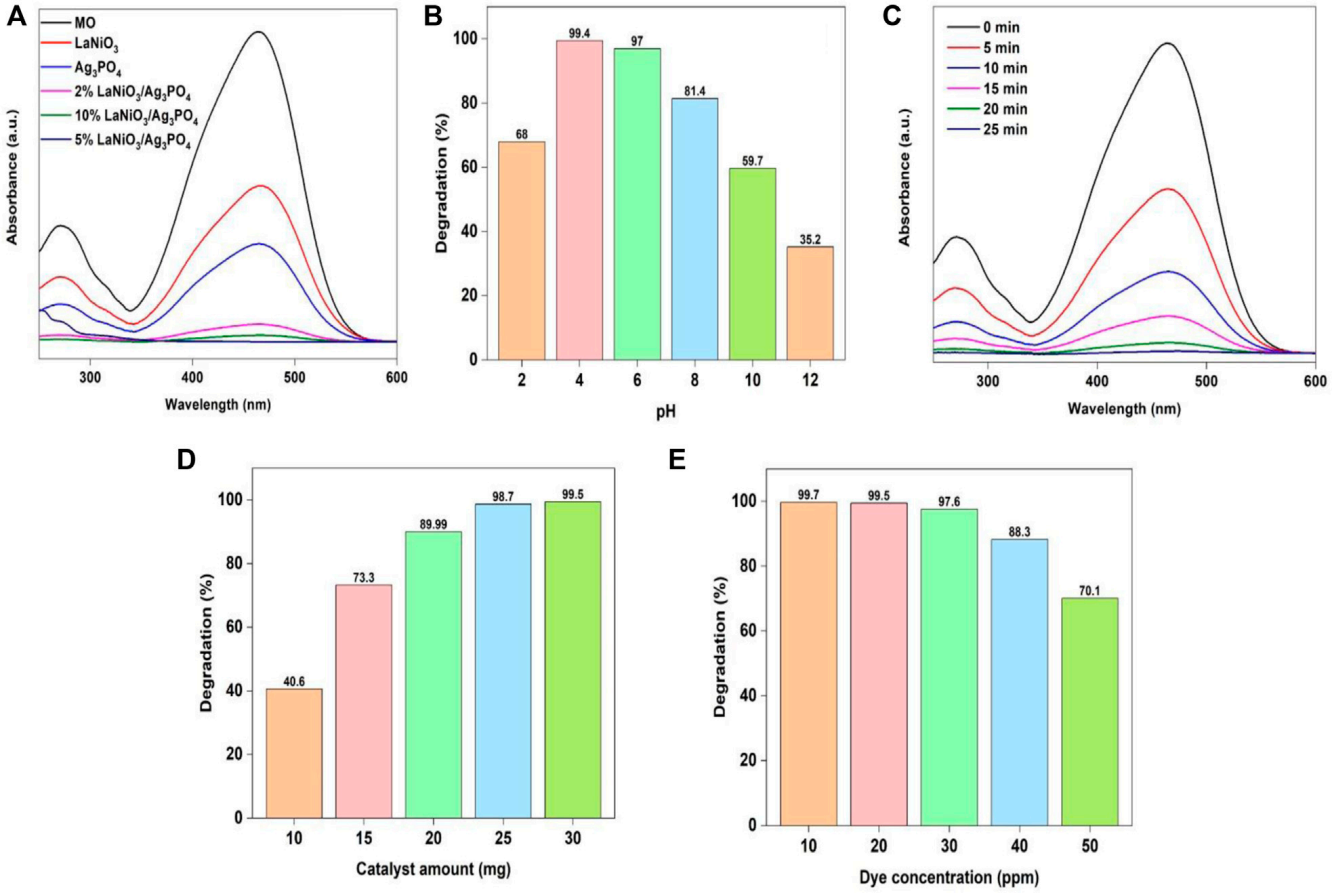
Change of MO degradation efficiency with variable **(A)** type of catalyst, **(B)** pH **(C)** time, **(D)** catalyst amount **(E)** dye concentration. Reaction conditions: Light Source: 100 watts LED, Intensity: 40 k Lux, temperature: 25°C, Pressure: 1 atm.

The photocatalytic activity of as-prepared samples for MO degradation was investigated with UV-Vis spectrophotometry, by using 25 mg of the each photocatalyst and 30 ml of MO solution (30 ppm). The resultant solutions were exposed to light for 25 min with continuous stirring. The most appropriate catalyst was selected based on their performance for dye degradation. The absorption spectrum of MO from photocatalytic studies was observed between the wavelength ranges from 200 to 600 nm. After some time, the strength of the MO distinctive peak began to decrease. The activity was increased by increasing the content of LaNiO_3_ in the LaNiO_3_/Ag_3_PO_4_ composite up to 5% but it decreased by further increasing the LaNiO_3_ content possibly due to agglomeration (Figure 7A). The results demonstrate that the 2% LaNiO_3_/Ag_3_PO_4_ and 10% LaNiO_3_/Ag_3_PO_4_ composite showed higher activity than pure LaNiO_3_ and Ag_3_PO_4_ but lower than 5% LaNiO_3_/Ag_3_PO_4_ composite. Therefore, 5% LaNiO_3_/Ag_3_PO_4_ composite was found to be suitable photocatalyst for the degradation of MO.

Further investigations were carried out to check the influence of pH on MO photodegradation. The photodegradation experiment was conducted out under the conditions: (Irradiation time: 25 min, amount of catalyst: 25 mg, solution’s pH: 2–12, concentration of MO 30 mg/L).

Figure 7B shows that the percentage efficiency for the removal of MO dye with 5%LaNiO_3_/Ag_3_PO_4_ is highest at pH 4. Because pH affects multiple variables, including ionization state of the photocatalyst and dye, ease of producing hydroxyl radicals, and catalyst agglomeration, it is referred to as a complicated parameter (Akpan and Hameed, 2009). Because photocatalytic reaction takes place predominantly on the surface of photocatalyst, the surface parameters have a significant effect on the performance of catalyst. Surface of the photocatalyst is positively charged in acidic conditions, and azo structure of methyl orange is also transformed into quinoid structure in acidic conditions. The quinoid structure of Methyl Orange is easy to degrade than azo structure as reported in literature (Basahel et al., 2015). Acidic condition promoted the degradation reaction catalyzed by 5%LaNiO_3_/Ag_3_PO_4_. Electrostatic interactions among the dye molecules and the surface of catalyst are also important in the degradation process. The increased efficiency in acidic conditions is explained by the anionic character of MO and absorption of H^+^ ions on the surface of catalyst. Under acidic conditions, the MO is easily absorbed on the surface of photocatalyst which is positively charged. In acidic media, removal of MO is more effective than in alkaline media (Mansouri et al., 2019).

The influence of reaction time on MO photodegradation was further investigated with the following conditions: (Irradiation time: 0–20 min, amount of catalyst: 25 mg, solution’s pH: 4, concentration of MO: 30 mg/L). Figure 7C shows that by extending the irradiation time, the efficiency of MO removal increases. Irradiation time of 20 min was selected for further study due to highest degradation efficiency for MO using the specified reaction conditions. To assess the amount of catalyst for the photocatalytic degradation of MO, the effect of catalyst dose was further investigated under the conditions: (Irradiation time: 20 min, amount of catalyst: 10–30 mg, solution’s pH: 4, concentration of MO 30 mg/L). The results showed that increasing the catalyst amount significantly increased capability of dye adsorption as well as rate of photodegradation. As shown in Figure 7D that increasing the catalyst dosage from 10 to 30 mg significantly enhances dye degradation efficiency. It is clearly seen that raising the photocatalyst amount increases the availability of active sites as well as illuminated area available for the degradation of extra dye molecules and adsorption. As a result, as the catalyst dosage was increased, the rate of photodegradation rose.

To investigate the influence of dye concentrations on photodegradation reaction, the photodegradation experiment was conducted under following conditions: (Irradiation time: 20 min, amount of catalyst: 25 mg, solution’s pH: 4, concentration of MO: 20–60 mg/L). A photodegradation process was shown to decrease as dye concentration increased, as illustrated in Figure 7E. This decrease in the rate of degradation with increasing dye concentration may be ascribed to the higher dye concentration reducing the active catalyst due to saturation effect. Furthermore, increasing the concentration of dye would enhance dye molecules adsorption on the photocatalyst surface and avoid the adsorption of oxygen and hydroxyl ions on the surface of photocatalyst for photodegradation.

### Degradation kinetics

**FIGURE 8:**
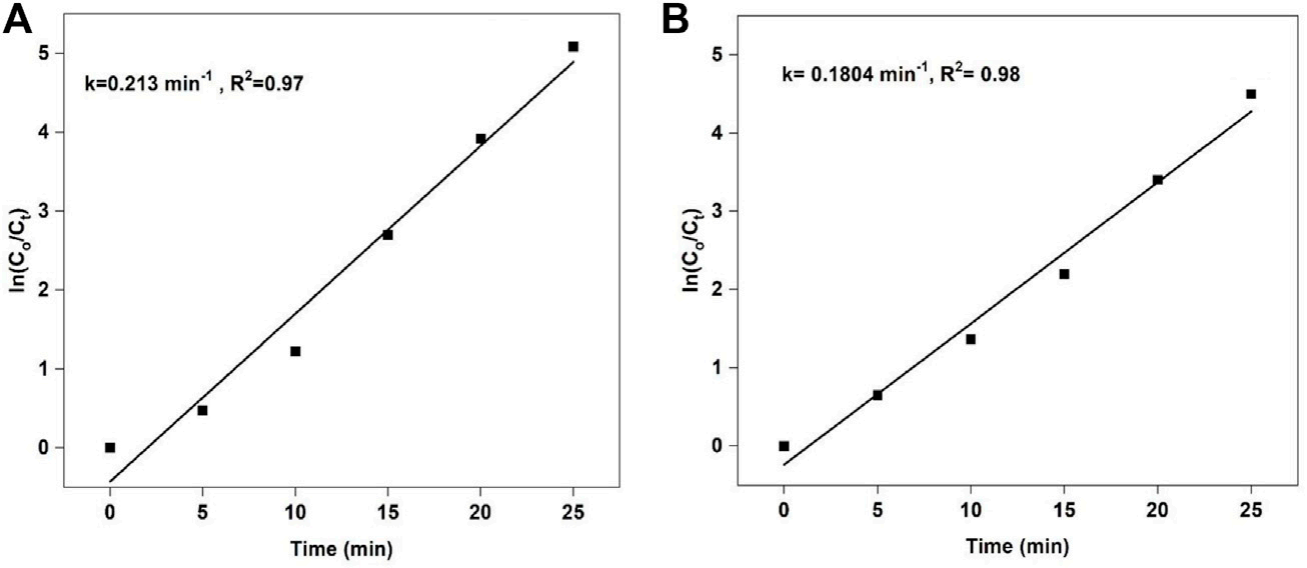
Pseudo first order kinetics of RhB **(A)** k = 0.2130 ± 0.0126, MO **(B)** k = 0.1804 ± 0.0113 Reaction conditions: Light Source: 100 watts LED, Intensity: 40 k Lux, temperature: 25°C, Pressure: 1 atm. Catalyst amount: 25 mg, Dye concentration: 30 mg, pH: Neutral.

To find the order and rate constant of degradation reaction, we have studied the degradation kinetics. The degradation reaction of rhodamine B by 5%LaNiO_3_/Ag_3_PO_4_ composite follows pseudo 1st order kinetic having rate constant 0.213 min^−1^. Whereas, the methyl orange degradation reaction by 5%LaNiO_3_/Ag_3_PO_4_ composite also shows pseudo 1st order kinetics with a rate constant of 0.180 min^−1^ as shown in Figures 8A,B).

### Possible mechanism for the photocatalytic dyes degradation

**FIGURE 9:**
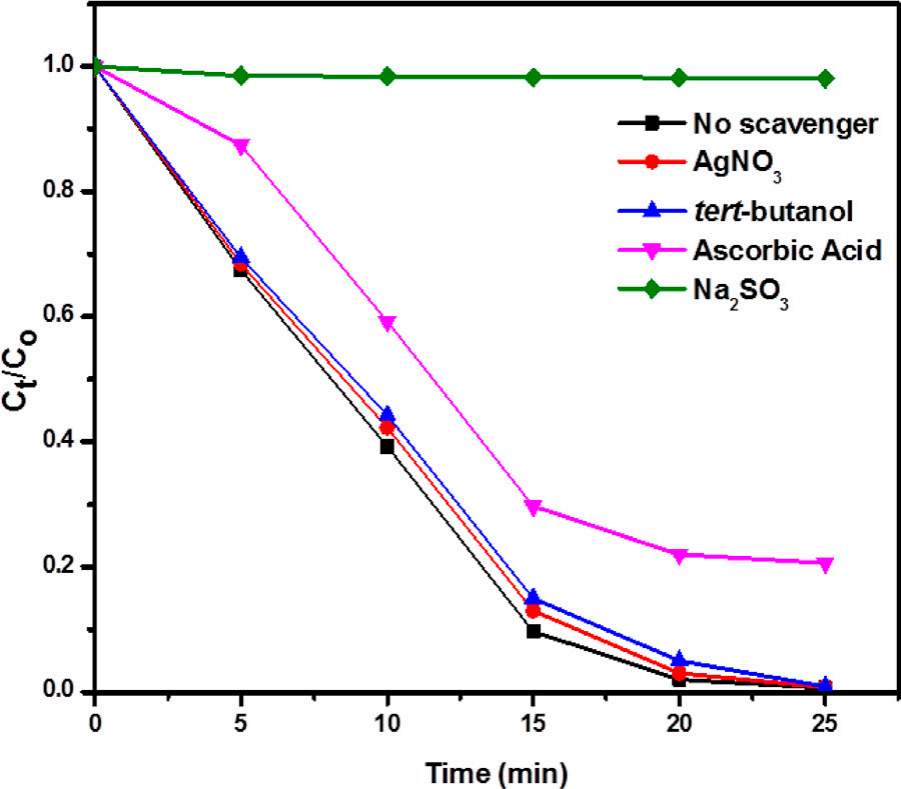
The effect of different scavengers on dye degradation of RhB. Reaction conditions: Light Source: 100 watts LED, Intensity: 40 k Lux, temperature: 25°C, Pressure: 1 atm. Catalyst amount: 25 mg, Dye concentration: 30 mg.

In order to understand the mechanism for the degradation of RhB and MO, the effect of different scavengers on RhB and MO degradation was carried out. Figure 9 shows that photocatalytic activity is completely inhibited by using hole scavenger sodium sulfite, but it remains almost unchanged by using electron scavenger (AgNO_3_). It means that holes play a crucial role in the degradation of RhB and MO. In the photodegradation system, the superoxide anion radical (O_2_^**·−**^) radicals are effective reactive oxygen species, which are produced by the reduction of O_2_ molecules adsorbed on the catalyst surface by the photogenerated electrons (Luo et al., 2019). Photocatalytic activity slightly changed by adding superoxide anion radical scavenger ascorbic acid while it remains unchanged by adding hydroxyl radical scavenger *tert*-butanol (TBA). So, superoxide anion radicals also play a role in the dye degradation of RhB and MO.

**FIGURE 10:**
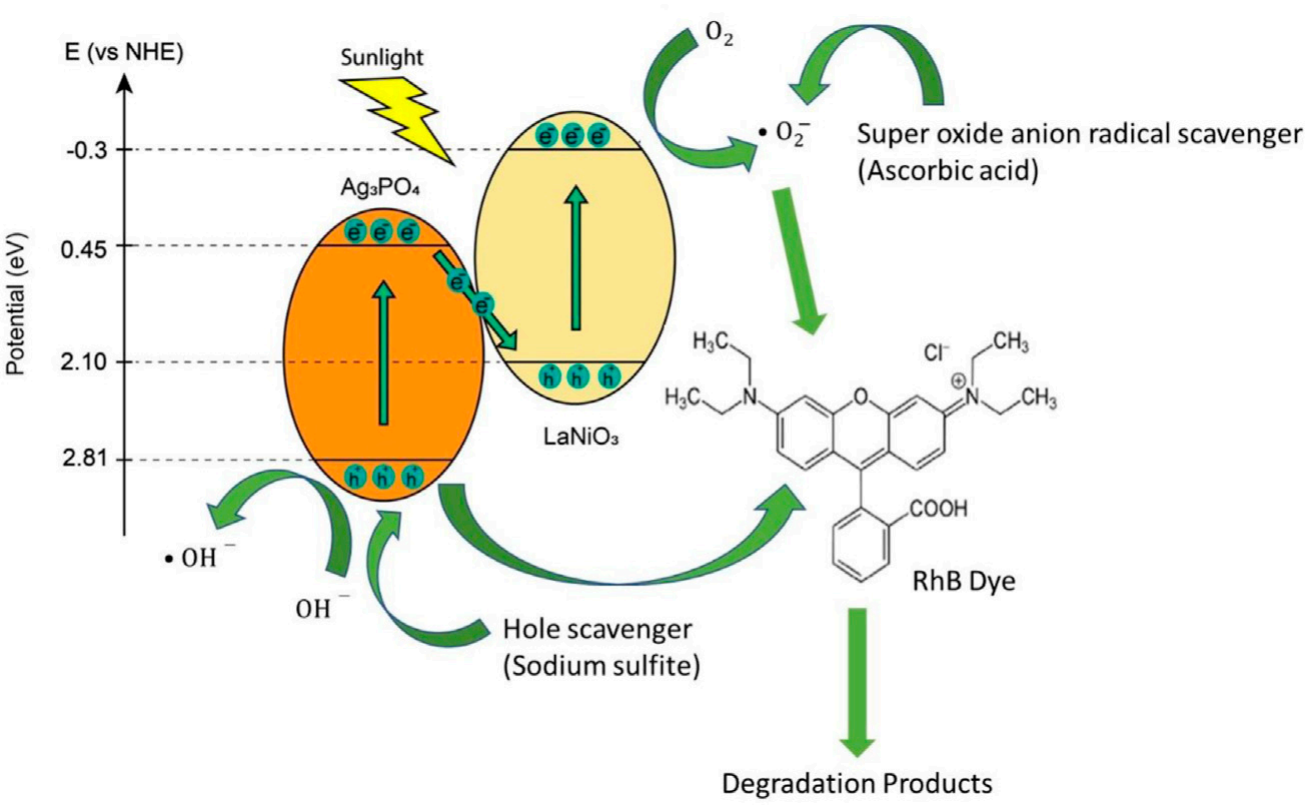
The possible mechanism for dye degradation.

Energy band positions of LaNiO_3_ and Ag_3_PO_4_ are shown in the Figure 10. The valence band positions of LaNiO_3_ and Ag_3_PO_4_ are 2.10 eV and 2.81 eV while conduction band positions are −0.3 eV and 0.45 eV respectively. Here, Z-Scheme heterostructure is formed between LaNiO_3_ and Ag_3_PO_4_. When the light falls on the surface of the photocatalyst, electrons from valence band of Ag_3_PO_4_ move towards conduction band of LaNiO_3_. Therefore, holes and electrons are formed (Ge et al., 2012; Khalid et al., 2020).

$$hv+Photocatalyst\to h^{+}+e^{-}$$

whereas holes react with the hydroxyl ions to form hydroxyl radicals.

$${OH}^{-}+h^{+}\to \cdot OH$$

Superoxide anion radicals are produced by the reduction of O_2_ molecules adsorbed on the catalyst surface by the photogenerated electrons.

$$O_{2}+e^{-}\to \;\cdot O_{2}^{-}$$

These generated radicals participated in the photocatalytic reduction and oxidation reactions to degrade dyes.

Scavenger results showed that mainly holes are responsible for the degradation of dyes. Therefore, the dyes adsorbed at the surface of catalyst degradation achieved by direct hole oxidation by catalyst suspension (Ge et al., 2012).

$$Pollutant+h^{+}\to DegradationProducts$$

### Stability of the photocatalyst

**FIGURE 11:**
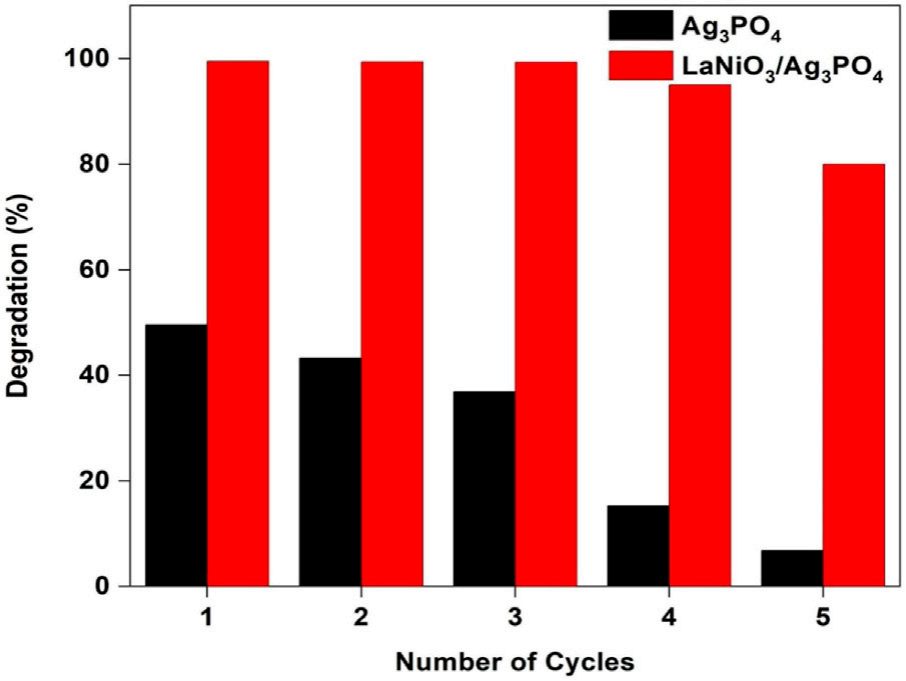
The stability of the photocatalyst with the number of cycles.

**FIGURE 12:**
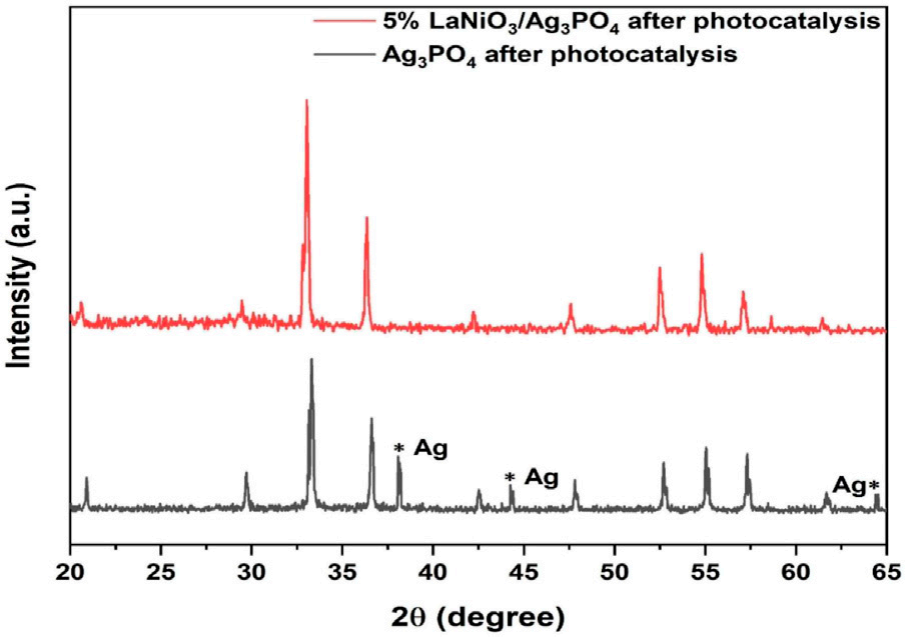
Powder XRD pattern of Ag_3_PO_4_, 5% LaNiO_3_/Ag_3_PO_4_ composite after photocatalysis.

The stability of the catalyst is also important for its practical applications. In order to investigate the stability of the photocatalyst, recycle experiments were carried out for pure Ag_3_PO_4_ and 5%LaNiO_3_/Ag_3_PO_4_. After each cycle the catalyst is washed with water/ethanol to remove any adsorbed dyes on the surface. Figure 11 shows that pure Ag_3_PO_4_ is not stable and shows catalyst degradation even up to 2^nd^ cycle, whereas 5%LaNiO_3_/Ag_3_PO_4_ is highly stable up to five cycles with degradation efficiency greater than 85%. XRD spectra of 5% LaNiO_3_/Ag_3_PO_4_ composite after five cycles were also recorded to check the stability of photocatalysts. Figure 12 shows that pure Ag_3_PO_4_ is decomposed to metallic Ag. While no signal from metallic Ag was observed with the 5% LaNiO_3_/Ag_3_PO_4_ composite, which confirming a higher stability of 5% LaNiO_3_/Ag_3_PO_4_ composite as compared to pure Ag_3_PO_4_.

### Comparison of dyes degradation from previously reported studies

Photodegradation is proved to be a very efficient method for the removal of RhB from water. In the past, a lot of work has been done in this regard. Different Ag_3_PO_4_ based composites, including Ag_3_PO_4_@GO, Ag_3_PO_4_/WO_3_, Ag_3_PO_4_/Ag, Ag_3_PO_4_/ZnO, Ag_3_PO_4_/N-TiO_2_, Ag_3_PO_4_/BiVO_4_, AgBr/Ag_3_PO_4_, Ag_2_MoO_4_/Ag_3_PO_4_, have been prepared and applied for visible light photocatalytic degradation of rhodamine B. These composites showed lower photocatalytic efficiency than recent study. Perovskite LaNiO_3_/Ag_3_PO_4_ photocatalyst showed the highest catalytic activity for the degradation of 50 ppm RhB solution in just 20 min, with rate constant of 0.213 min^−1^ using small amount of catalyst 25 mg as shown in Table 1.

**TABLE 1:** Comparison of RhB photodegradation by various catalysts from the literature.

Catalyst	Catalyst amount (mg)	Dye amount (mg/L)	Degradation time (min)	Degradation efficiency (%)	Rate constant (min^−1^)	References
Ag_3_PO_4_@GO	50	6	60	99	–	Liu et al. (2018)
Ag_3_PO_4_/WO_3_	40	5	30	97	–	Zhang et al. (2014)
Ag_3_PO_4_/Ag	100	10	90	98	–	Huang et al. (2015)
Ag_3_PO_4_/ZnO	20	10	30	93	0.0895	Dong et al. (2014)
LaNiO_3_	10	10	120	53	0.006	Ghafoor et al. (2021)
Ag_3_PO_4_/N-TiO_2_	20	10	120	99	0.0194	Khalid et al. (2020)
Ag_3_PO_4_/BiVO_4_	100	10	30	92	0.088	Qi et al. (2016)
Ag_2_MoO_4_/Ag_3_PO_4_	50	10	12	97	0.3591	Cao et al. (2017)
AgBr/Ag_3_PO_4_	100	10	7	99	–	Wang et al. (2013)
Bi_4_Ti_3_O_4_/Ag_3_PO_4_	20	5	30	99	0.1789	Zheng et al. (2017)
g-C_3_N_4_/Ag_3_PO_4_	100	10	10	96	–	He et al. (2014)
Ag_3_PO_4_/CdWO_4_	100	10	5	99	0.71	Zhang et al. (2020)
CNT/Ag_3_PO_4_	75	10	12	92.4	0.207	Xu et al. (2014)
LaNiO_3_/Ag_3_PO_4_	25	50	20	90.5	0.213	Present Study

Similarly various photocatalysts have been used for MO photodegradation in the literature. Different Ag_3_PO_4_ based composites including AgBr/Ag_3_PO_4_, Ag_3_PO_4_/TiO_2_, Ag_3_PO_4_/g-C_3_N_4_, Ag_3_PO_4_/BiPO_4_, Ag_3_PO_4_/GO, Ag_3_PO_4_/Nb_2_O_5_, Ag_3_PO_4_/AgI, Ag/Ag_3_PO_4_, Ag_3_PO_4_/BiOI, Ag_3_PO_4_/MoS_2_ have been synthesized and used for visible light photocatalytic degradation of MO. The present studies shows the superior catalytic efficiency in terms of catalyst amount i.e., 25 mg, MO degradation concentrations (50 mg/L), irradiation time (20 min) degradation efficiency of 99.5%, with rate constant of 0.1804 min^−1^ as shown in Table 2.

**TABLE 2:** Comparison of MO photodegradation by various catalysts from the literature.

Catalyst	Catalyst amount (mg)	Dye amount (mg/L)	Degradation time (min)	Degradation efficiency (%)	Rate constant (min^−1^)	References
AgBr/Ag_3_PO_4_	100	10	40	95.1	-	Cao et al. (2012)
Ag_3_PO_4_/TiO_2_	40	10	60	95.5	0.052	Liu et al. (2019)
Ag_3_PO_4_/g-C_3_N_4_	100	20	30	94	-	Hu et al. (2020)
Ag_3_PO_4_	50	5	30	58	-	Ge, (2014)
LaNiO_3_	400	10	300	74.9	0.1520	Li et al. (2010)
Ag_3_PO_4_/BiPO_4_	100	10	60	98	0.106	Lin et al. (2013)
Ag_3_PO_4_/GO	80	20	50	86.4	0.0394	Chen et al. (2013a)
Ag_3_PO_4_/Nb_2_O_5_	500	10	60	96	0.073	Osman et al. (2021)
Ag_3_PO_4_/AgI	100	20	18	96.9	-	Chen et al. (2013b)
Ag/Ag_3_PO_4_	300	20	12	99	0.215	Liu et al. (2012)
Ag_3_PO_4_/rGO	20	10	210	92	0.010	Dong et al. (2013)
Ag_3_PO_4_/BiOI	50	10	50	98	0.109	Wang et al. (2015)
Ag_2_S/Ag_3_PO_4_	35	10	120	99	0.0338	Tian et al. (2017)
Ag_3_PO_4_/PbBiO_2_Br	100	20	50	98	–	Xia et al. (2019)
Ag_3_PO_4_/NiFe_2_O_4_	20	10	30	96.8	0.12	Huang et al. (2018)
Ag_3_PO_4_/MoS_2_	30	20	120	98.2	0.0654	Zhu et al. (2016)
LaNiO_3_/Ag_3_PO_4_	25	50	20	99.4	0.1804	Present Study

## Conclusion

Highly efficient perovskite LaNiO_3_/Ag_3_PO_4_ photocatalyst was successfully prepared by the facile hydrothermal method. Firstly, LaNiO_3_ was prepared and then composite formed with Ag_3_PO_4_ by hydrothermal synthesis. All the synthesized photocatalysts have shown excellent results for the degradation of rhodamine B and methyl orange in a short interval of time, and almost complete degradation efficiency was achieved. Among all synthesized photocatalysts, 5% LaNiO_3_/Ag_3_PO_4_ composite has been proved to be an excellent photocatalyst for the photodegradation of RhB and MO.

The average crystallite size of silver phosphate was successfully reduced from 86.52 nm to 58.19 nm by incorporating pure phase lanthanum nickelate into silver phosphate. The photooxidation activity and stability of Ag_3_PO_4_ were also enhanced by the introduction of LaNiO_3_ in Ag_3_PO_4_ heterojunction. Complete photodegradation of 50 mg/L solutions of both dyes using 25 mg of 5% LaNiO_3_/Ag_3_PO_4_ photocatalyst were observed in just 20 min. Photodegradation of both dyes RhB and MO follows pseudo-first-order kinetics with rate constants of 0.213 and 0.1804 min^−1^, respectively. It was observed that photocatalytic activity was completely inhibited by using a hole scavenger, which means that holes are mainly responsible for the degradation of both dyes. Photocatalyst showed the highest stability up to five cycles with degradation efficiency greater than 85%.

## Data Availability

The original contributions presented in the study are included in the article/Supplementary Material, further inquiries can be directed to the corresponding authors.
